# Supplementary material for: Amplified Vasodilatation within the Referred Pain Zone of Trigger Points Is Characteristic of Gluteal Syndrome—A Type of Nociplastic Pain Mimicking Sciatica
Source: J Clin Med. 2021 Nov 2;10(21):5146. doi: 10.3390/jcm10215146 (PMC8584656; doi:10.3390/jcm10215146)
Supplement: Supplementary file 1 [file jcm-10-05146-s001.zip › chapter S1.pdf]

Procedure in MATLAB

A general scheme of the procedure for automated processing of the thermographic images obtained during the Minimally Invasive Procedure and an analysis of all the data recorded during the examination stage are shown in Fig. A. The procedure involved the following steps: (1) the manual generation of mask images described as *Manual procedure A*; (2) the conversion of mask images to the matrix described as *MATLAB procedure B*; and (3) the calculation of features and measures performed in the MATLAB environment described as *MATLAB procedure C*.

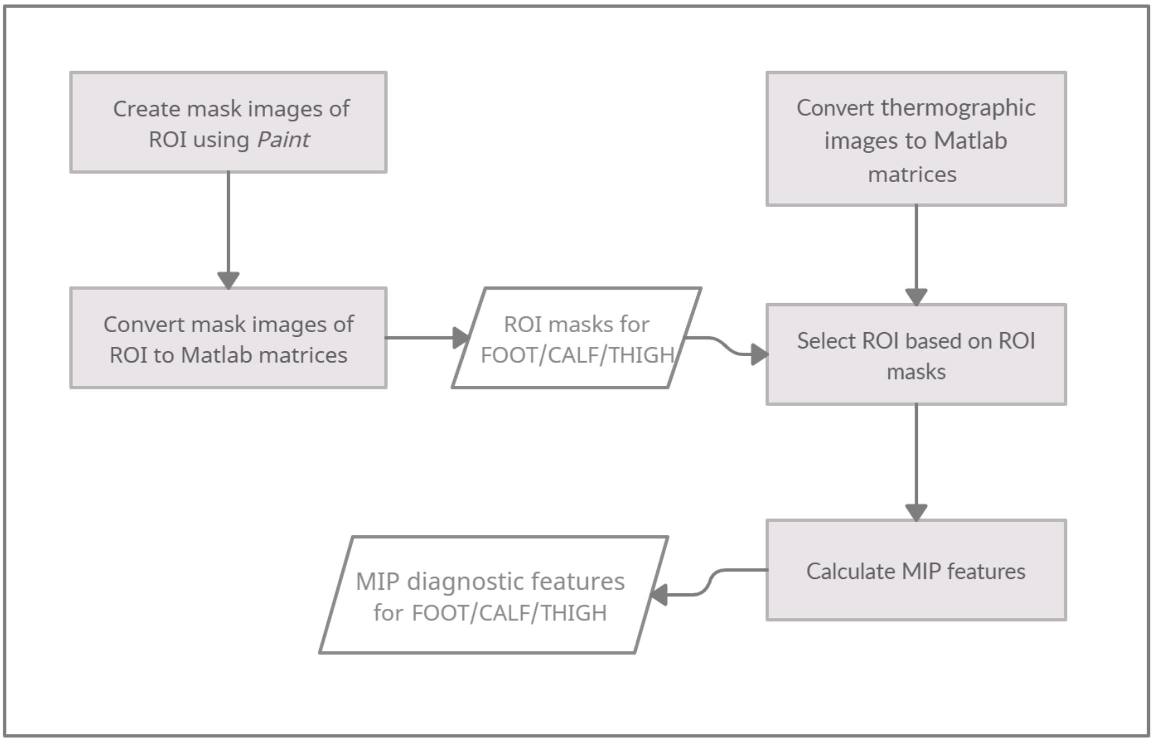

Fig. A. A general flow chart of the calculation procedures for features and measures performed in the MATLAB environment.

Manual Procedure A

Before the automated data analysis was performed in the MATLAB environment, images with the marked ROI (region of interest)—i.e., the masks—were prepared manually. The masks were defined anatomically based on the thigh, calf, and foot. This procedure was performed for each patient separately using the *Paint* editing software. Automatic mask imposition was impossible due to the different sizes of the lower limbs between individual patients and the different positions of the observed lower limbs among different subjects. To date, no procedure has been

developed to automatically detect the foot, calf, and thigh in a thermographic image. As a result of this manual procedure, FOOT, CALF, and THIGH masks were developed. A sample visualization of the masks is shown in Fig. B.

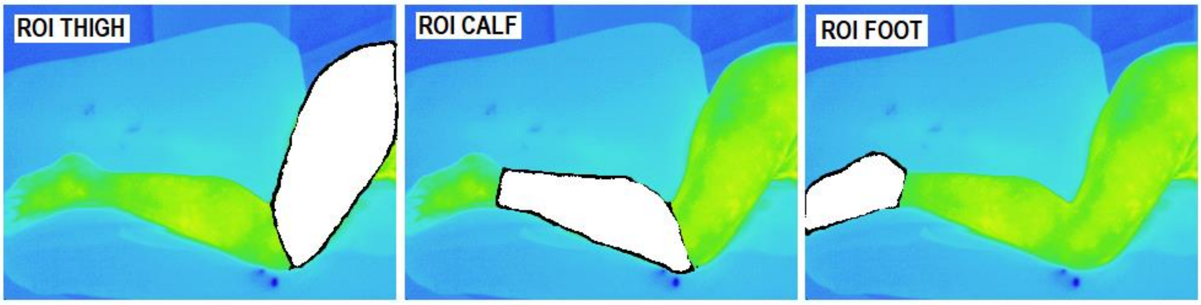

Fig. B. The result of manual procedure A: ROI mask creation for FOOT, CALF, and THIGH.

MATLAB procedure B

Thermograms with masks were loaded as BMP files into the MATLAB environment to convert them into matrices, which was necessary for further analysis. The procedure for thermogram processing was as follows:

1) Conversion from a true RGB color image into grayscale using the *rgb2gray* function. This task was performed by eliminating the hue and saturation information while retaining the luminance. The grayscale values were calculated according to the following formula: 0.299 * R + 0.587 * G + 0.114 * B, where R is the red component, G is the green component, and B is the blue component. As a result, a 320x240 matrix of values in a range from 0 to 255 UINT8 was created.
2) Conversion of the UINT8 values to the floating-point format using the *double* function.
3) Reduction of the shadows by eliminating all values but the 255 value that corresponds to the ROI.
4) Division of the matrix by 255 to create a '0'/'1' matrix, where '1' corresponds to the number of pixels within the ROI.

Upon the completion of thermogram processing using MATLAB, matrices for the three ROIs were obtained, as visualized in Fig. C.

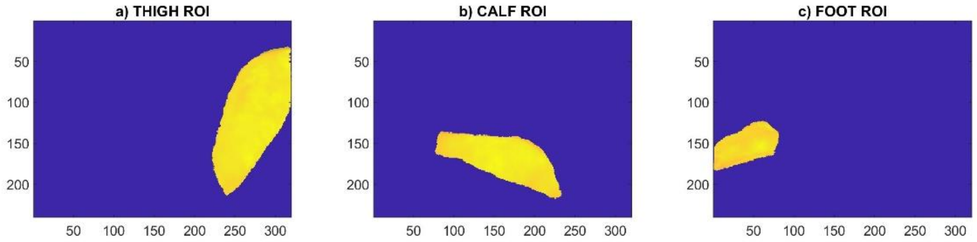

Fig. C. Visualization of MATLAB procedure B: The original thermogram for a random subject (a); the selected ROI for FOOT (b), CALF (c), and THIGH (d).

MATLAB Procedure C

The MIP thermographic images of each patient were exported as text files. The images were then loaded into the MATLAB environment and stored as a 320x240 matrix. Each element of the matrix corresponds to a temperature value rounded to one decimal place. The matrices were then multiplied by mask matrices for the three areas of THIGH, CALF, and FOOT. The mask matrices were calculated according to MATLAB procedure B, and the obtained matrices were saved for further calculations.

This step was followed by a data cleaning procedure, including the deletion of patient data if the measurement took less than 15 minutes (due to a recording error of the thermal camera), or if the limb was significantly moved during the measurement, as such movement would make the automated calculation of the desired features and measures impossible.

Measurements and how they were calculated

The evaluation of MIP diagnostic parameters included calculation of the AURP, i.e., the percentage of the area with a temperature response, calculated separately for vasodilatation and vasoconstriction (type I for vasodilatation $AURP_{Tmax}$ and type II for vasoconstriction $AURP_{Tmin}$). The second analyzed parameter was the level of change in the mean temperature of the area (ΔT_{avr}).

The following procedure was used for calculating features and measures:

1) Calculation of the area with the AURP temperature response in the ROI
   a. Calculate the values of the minimum T_{min} and maximum T_{max} temperature in the first thermogram at time $t_0 = 0$ sec.

$$\bigvee T_{px} \in \{1, 2, \dots, n\} \wedge t = t_0,\ T_{max} = \max(T_{px1}, T_{px2}, \dots, T_{pxn})$$
$$\bigvee T_{px} \in \{1, 2, \dots, n\} \wedge t = t_0,\ T_{min} = \min\left(T_{px1}, T_{px2}, \dots, T_{pxn}\right).$$

b. Calculate the area of the ROI surface A_{ROI} for each patient as the sum of non-zero pixels in the thermogram according to the following formula:

$$\bigvee T_{px} > 0\,, A_{ROI} = \sum T_{px}$$

where T_{px} is the temperature value at pixel *px* within the ROI.

c. Calculate the percentage of the area with a temperature equal to T_{min}, A_{min} and equal to T_{max}, A_{max} at time t_0 according to the following formula:

$$\bigvee T_{px} : T_{px} = T_{max} \ \wedge \ t = t_0, A_{max} = \frac{\sum T_{px}}{A_{ROI}} * 100\ \%$$

$$\bigvee T_{px} : T_{px} = T_{min} \ \wedge \ t = t_0, A_{min} = \frac{\sum T_{px}}{A_{ROI}} * 100\ \%.$$

d. Calculate the percentage of the area with a temperature greater than or equal to T_{max} for $AURP_{Tmax}$ according to the following formula:

$$\bigvee T_{px} : T_{px} \geq T_{max} \ \wedge \ t > t_0, AURP_{Tmax} = \frac{\sum T_{px}}{A_{ROI}} * 100\ \%.$$

e. Calculate the percentage of the area with a temperature lower than or equal to T_{min} for $AURP_{belowTmin}$ according to the following formula:

$$\bigvee T_{px} : T_{px} \leq T_{min} \ \wedge \ t > t_0, AURP_{Tmin} = \frac{\sum T_{px}}{A_{ROI}} * 100\ \%.$$

2) Calculate the change in the mean temperature of the ROI

a. Calculate the value of the arithmetic mean temperature T_{avg_tx} in subsequent thermograms at time t_x, where x={0, 3, 6, 9, …, 900} sec.

b. Calculate the value of temperature changes ΔT_{avr_tx} at t_x moments as the difference between the average temperature values at successive time points and the value at t_0 according to the following formula: $\Delta T_{avr\_tx} = T_{avg\_tx} - T_{avg\_t0}$.
