# Supplementary figures and images for: Amplified Vasodilatation within the Referred Pain Zone of Trigger Points Is Characteristic of Gluteal Syndrome—A Type of Nociplastic Pain Mimicking Sciatica
Source: J Clin Med. 2021 Nov 2;10(21):5146. doi: 10.3390/jcm10215146 (PMC8584656; doi:10.3390/jcm10215146)

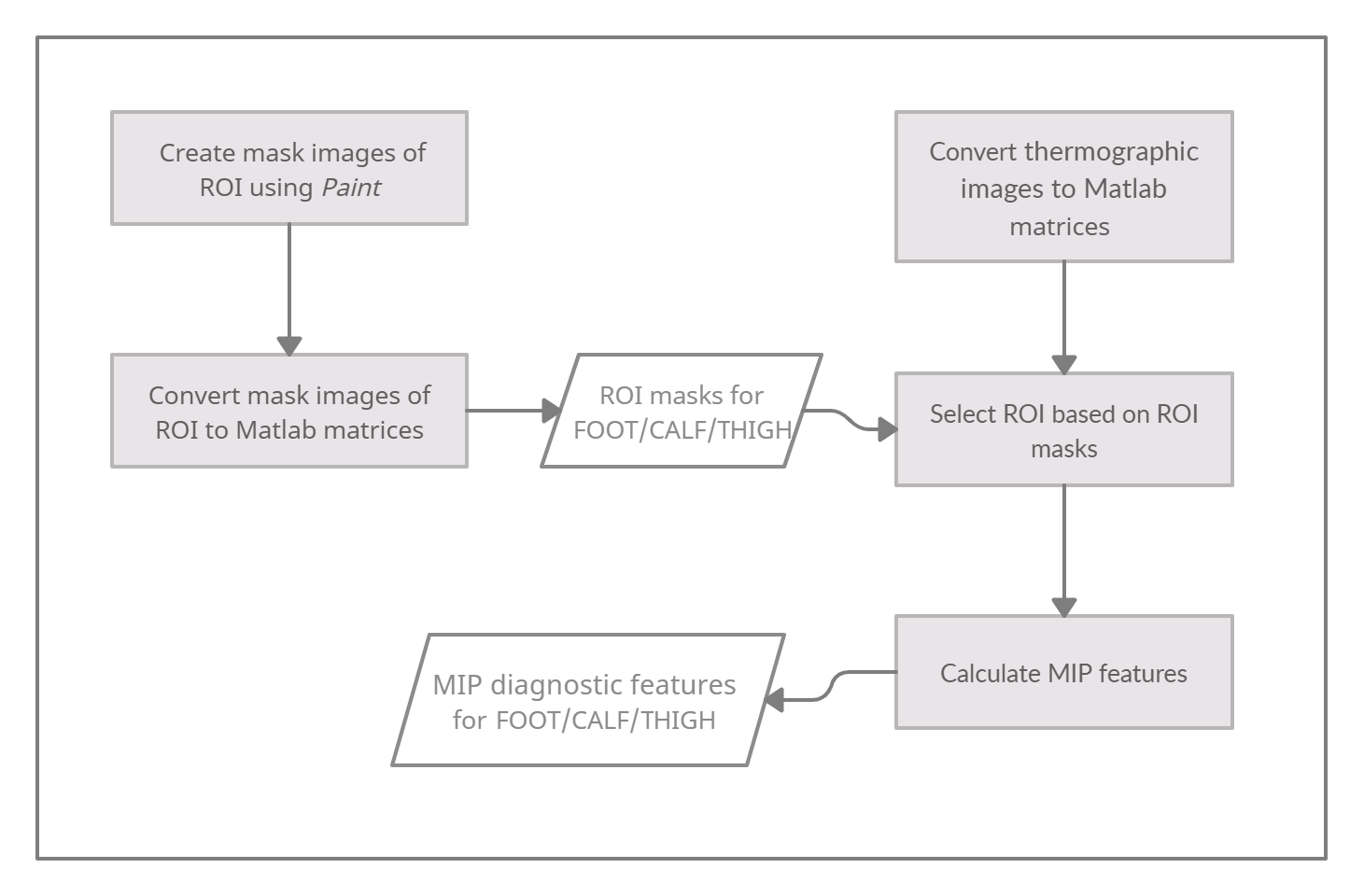

Supplement: Supplementary file 1 [file jcm-10-05146-s001.zip › Fig.A_chapterS1 (2).jpg]

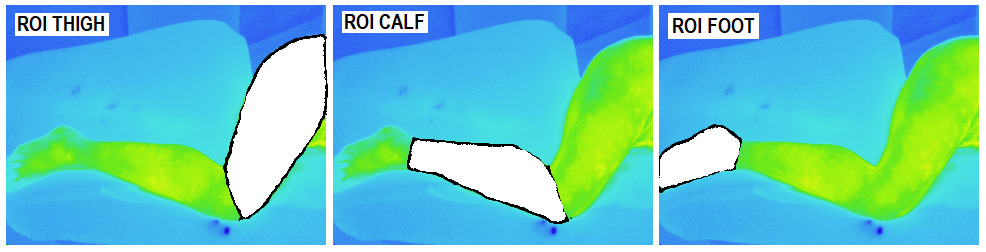

Supplement: Supplementary file 1 [file jcm-10-05146-s001.zip › Fig.B_chapterS1 (1).jpg]

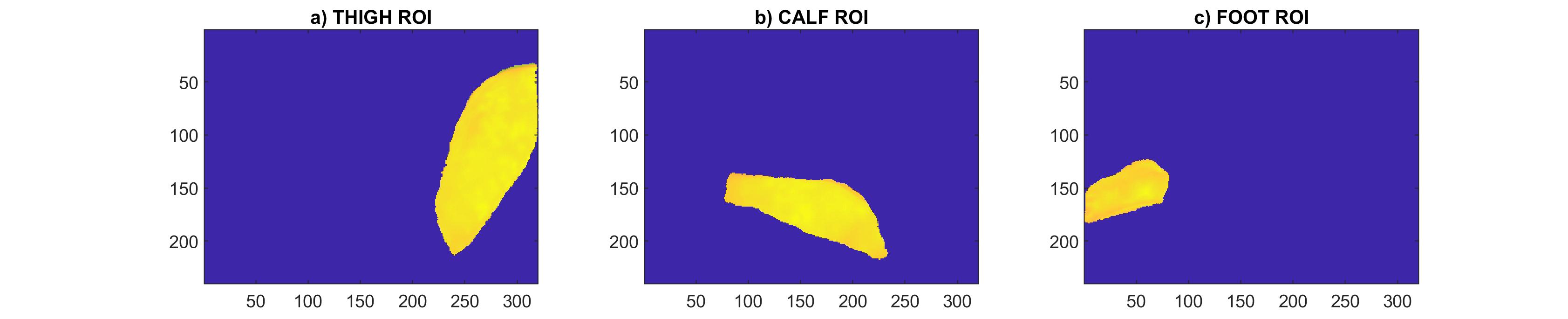

Supplement: Supplementary file 1 [file jcm-10-05146-s001.zip › Fig.C_chapterS1 (3).jpg]
